# A Case of Native Joint Septic Arthritis Due to Histoplasma Capsulatum in a Patient on Adalimumab

**DOI:** 10.7759/cureus.107014

**Published:** 2026-04-14

**Authors:** Seth Conley, Anmol Baidwan, Moumita Sarker

**Affiliations:** 1 Internal Medicine, Mary Washington Healthcare, Fredericksburg, USA; 2 Infectious Disease, Mary Washington Healthcare, Fredericksburg, USA

**Keywords:** adalimumab (humira), endemic mycoses, histoplasma capsulatum, immunocompromised patient, septic arthritis of knee

## Abstract

Disseminated histoplasmosis is primarily a disease of the immunosuppressed, with clinically significant symptoms most commonly observed in the lungs, intra-abdominal organs, or central nervous system. We present a case of native joint septic arthritis due to *Histoplasma capsulatum* in an 88-year-old male on adalimumab for psoriasis with no other etiologies for immunosuppression. The patient presented with four weeks of right knee pain without inciting trauma. Synovial fungal cultures grew *Histoplasma capsulatum. *He underwent incision and drainage with anterior compartment synovectomy along with six months of oral itraconazole. Ultimately, the patient's infection resolved. Repeat cultures were negative, and he returned to his baseline physical activity. While recombinant monoclonal antibodies such as adalimumab have been revolutionary in the treatment of autoimmune diseases, this case highlights how infections may have atypical presentations and the importance of close infection surveillance for those on these medications.

## Introduction

*Histoplasma capsulatum *is a ubiquitous fungus found within the environment. It is most commonly found in Central America and the Ohio and Mississippi river valleys of the United States [1]. Thermally dimorphic, *Histoplasma* exists in a mold stage at environmental temperatures of 25°C or below, with transformation to yeast at 37°C [1,2]. Exposure typically occurs from soil disruption during daily activities in endemic areas. Inoculation occurs after inhalation of soil containing dormant mold spores known as conidia. Conidia enter the alveoli, where they are phagocytosed by respiratory macrophages. Once exposed to intracellular temperatures, it is able to transition from a dormant mold to a virulence-expressing yeast [3,4]. Phagocytosed yeast suppresses reactive oxygen within the macrophage via production of a unique superoxide dismutase [5]. CD4^+^ T lymphocytes suppress infection by initiating granuloma formation containing infected macrophages, thus preventing *Histoplasma *dissemination [5]. 

Primary histoplasmosis is a pulmonary disease with less than 1% of infections being clinically relevant and possessing symptoms attributable to* Histoplasma* [1]. If not spontaneously cleared, it possesses the ability to disseminate via lymphatic spread, where it lies dormant in macrophages [2-4]. Subsequent immunosuppression can lead to clinically significant disease reactivation that is sometimes fatal [3-5]. Disseminated disease can present in essentially any organ system, with the lungs, intra-abdominal organs, and the central nervous system being the most symptomatic sites [3]. In contrast, cases of septic arthritis of native joints are incredibly rare. This is likely due to the baseline lack of vasculature within the joint cartilage, effectively preventing initial seeding and posing a lack of access to vital nutrients for the fungus [6]. When articular infection does occur, it is usually in the setting of autoimmune arthropathies with reactivation occurring after the initiation of disease-modifying antirheumatic drugs or immunotherapy [7,8]. 

Fungal infections are a prevalent complication of immunotherapeutic agents, particularly tumor necrosis factor alpha inhibitors [9]. Of these infections, *Histoplasma* is the most common etiology [10]. While these medications have been revolutionary in the management of malignancies, inflammatory bowel disease, and autoimmune arthropathies, they cause a significantly increased risk for infections. Currently, the required infectious screening tests before initiating adalimumab are hepatitis B virus and tuberculosis [11]. Retrospective analysis has shown that serious infection is the most common reason for adalimumab discontinuation [12]. It has been proven that therapy can be resumed once *Histoplasma* infection has been treated without risk of recurrence [13]. Early recognition and high suspicion for invasive fungal disease in those treated with tumor necrosis factor alpha inhibitors may lead to decreased mortality and morbidity by safely resuming therapy after fungal eradication.

## Case presentation

**Figure 1:**
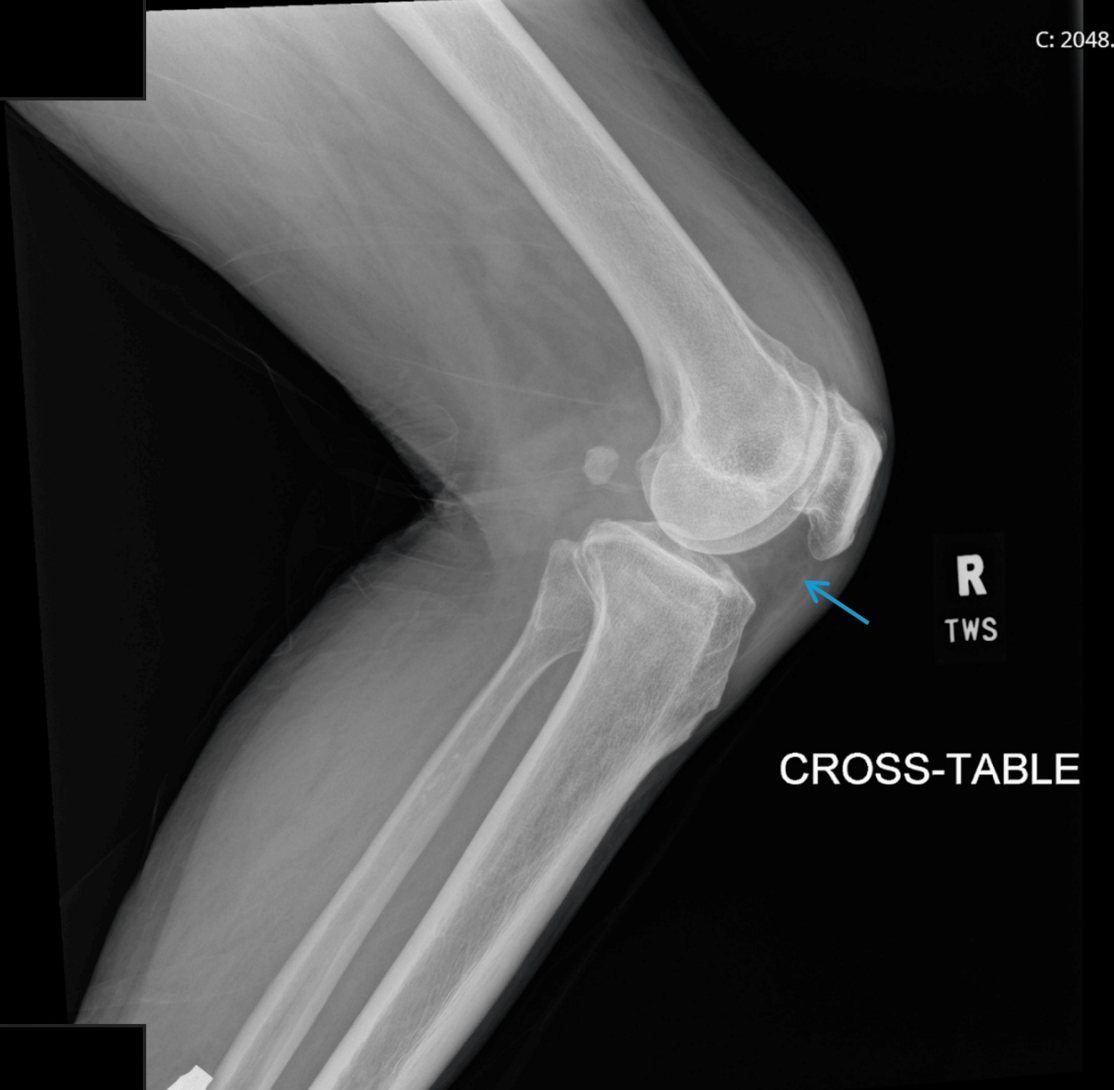
Initial X-ray in the emergency department showing a joint effusion

An 88-year-old Vietnamese male with a past medical history of benign prostatic hyperplasia, chronic constipation with recurrent small bowel obstruction, and psoriasis treated with adalimumab presented to the emergency department for four weeks of right knee pain, swelling, and warmth without any precipitating trauma. He denied any prior arthropathies to his knees or other joints. In the preceding two weeks, he reported decreased ambulation due to pain. He was afebrile with a temperature of 97°F, normal vitals, and expressed no other constitutional symptoms on presentation. On physical examination, he had tenderness to global palpation of the joint, minimal edema, and a full range of both active and passive motion with only mild discomfort. An X-ray of the right knee was performed, which showed no bony abnormalities but the presence of a possible joint and prepatellar effusion (Figure 1). 

Initial labs showed a slightly elevated CRP of 5.6 mg/L but no leukocytosis. All other labs were non-contributory. The patient was admitted for orthopedic evaluation of the knee. He underwent joint aspiration of the right knee, which showed red and cloudy synovial fluid, 48,000 red blood cells, and 31,796 nucleated cells, of which 73% were neutrophils. No crystals were noted. The Gram stain showed numerous polymorphonuclear leukocytes, but no organisms were observed. Given fluid analysis suggestive of septic arthritis, he was started on IV vancomycin, and infectious disease was consulted. The patient underwent incision and drainage and anterior compartment synovectomy three days after aspiration. Fungal, acid-fast bacilli, and bacterial cultures were collected. A broad-range bacterial polymerase chain reaction (PCR) was performed. Four days later, cultures remained negative, and PCR had not returned. In the setting of culture-negative septic arthritis, a peripherally inserted central catheter line was placed, and the patient was set up for three weeks of IV vancomycin and ceftriaxone until final cultures and PCR results returned. The patient was discharged on day seven of admission. 

**Figure 2:**
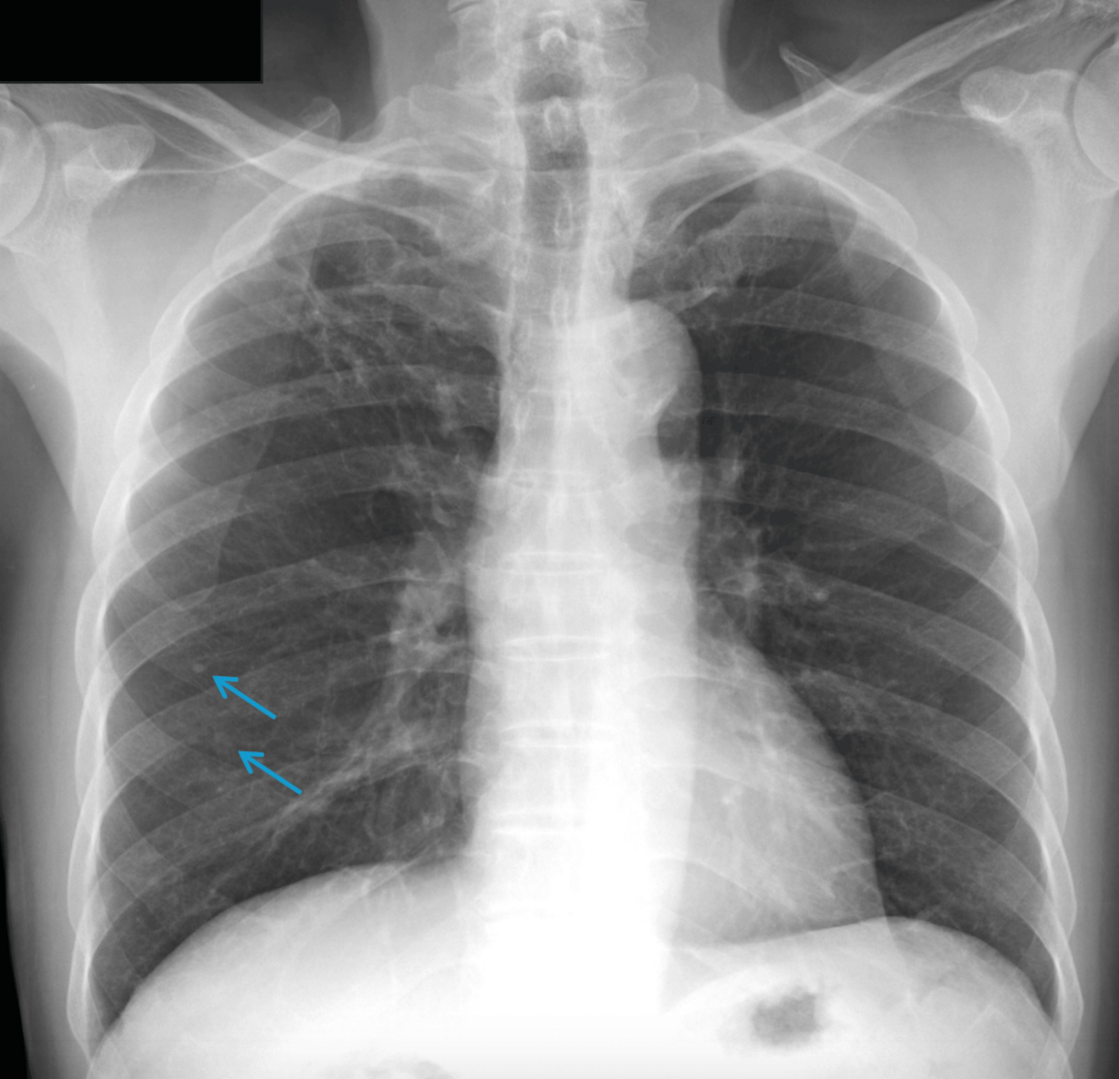
Pulmonary granulomas, likely indicative of prior Histoplasma infection, as identified by on service radiologist

One week after discharge, intraoperative synovial fungal culture was positive for mold per the preliminary laboratory report but without speciation. The patient and his family were notified of this finding by the infectious disease physician. He had no travel to the classically associated areas for *Histoplasma*, living primarily in Vietnam, then briefly in the southeastern seaboard of the United States. Antibiotics were discontinued, and IV voriconazole was initiated. Fungal blood cultures, urine *Histoplasma* antigen, urine *Blastomyces* antigen, cryptococcal serum antigen, Beta-D-Glucan (Fungitell), chest X-ray, HIV, T-helper cells, and immunoglobulin levels were ordered. Review of prior imaging was significant for pulmonary granulomas first noted five years ago. Repeat imaging was unchanged, with findings of calcified granulomas of the right lower lobe (Figure 2). Fungal blood cultures were negative. Beta-D-Glucan was uninterpretable due to an artifact. Immunoglobulin levels and T-helper cell analysis are presented in Table 1. HIV screen was negative. Urine *Blastomyces*, *Histoplasma*, and serum cryptococcal antigens were negative. Synovial fluid fungal culture reported final identification of *Histoplasma capsulatum*. The patient was then transitioned to oral itraconazole 200 mg twice a day to complete a total of six weeks of antifungal therapy. 

**Table 1:** Laboratory data obtained by infectious disease physician

Laboratory item	Patient values	Reference ranges and units
IgG	1615 mg/dL	600-1600 mg/dL
IgA	519 mg/dL	60-400 mg/dL
IgM	112 mg/dL	40-250 mg/dL
CD3	937 cells/mcL	Unestablished for the age group
CD4	570 cells/mcL	Unestablished for the age group
CD8	482 cells/mcL	Unestablished for age group
HIV 1/2 Ag Ab	Negative	Negative

**Figure 3:**
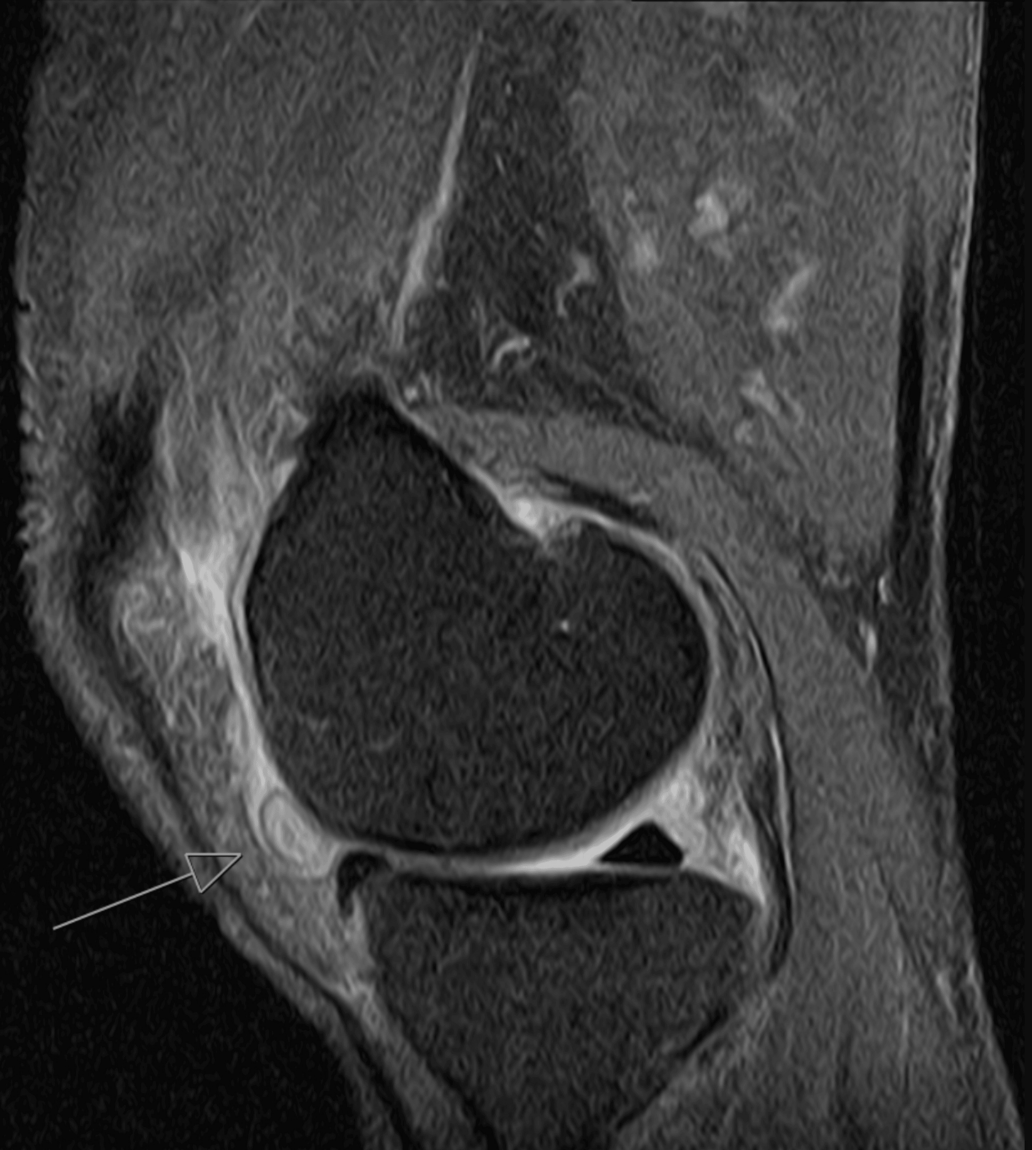
MRI of right knee obtained one month after completion of initial course of antifungal therapy showing mass-like synovial thickening, a sequela of Histoplasma capsulatum septic arthritis.

During his follow-up visit after completing this course of antifungal therapy, the patient continued to endorse right knee pain and swelling, despite the surgical and pharmacologic management. MRI of the knee was obtained and showed extensive synovitis and synovial thickening (Figure 3). Further surgical, pharmacological, and observational options were discussed with the patient and family, who ultimately elected to continue with a prolonged course of oral itraconazole for an additional four and a half months to make a total of six months of antifungal therapy. After these additional four and a half months, his knee pain and swelling nearly resolved, and he returned to his regular activity levels. Repeat Beta-D-Glucan, fungal cultures, and inflammatory markers (CRP and erythrocyte sedimentation rate) were all negative. A liver function panel was obtained to ensure there was no development of drug-induced hepatotoxicity and that it was within the normal range. The patient and his family wished to restart adalimumab therapy due to worsening psoriasis lesions, so he was recommended to continue low-dose itraconazole at 100 mg daily for an additional six months as secondary prophylaxis. Six months after resumption of adalimumab, the patient had no symptoms of prior septic arthritis and was noted to have improvement of his psoriatic lesions.

## Discussion

*Histoplasma capsulatum* is increasing in prevalence, particularly in areas that were historically not considered endemic for *Histoplasma*. Given this, geography alone should not be used to exclude histoplasmosis as a differential diagnosis [14]. Newfound understanding of fungal epidemiology has broadened clinical awareness. In response, the CDC has created statistical models predicting the likelihood of endemic* Histoplasma* both in the United States and globally [15]. The prevalence of immunomodulating therapies and histoplasmosis has shown a direct relationship over time [16]. Despite this, treatment guidelines have had little change until earlier this year, in 2025. Current Infectious Disease Society of America recommendations state that those with radiographic evidence of histoplasmosis (*Histoplasma* pulmonary nodules) and without evidence of active disease do not require treatment unless they are in a moderate-to-high risk group to develop disseminated or severe disease, which is reviewed in Table 2. Of relevance, those on immunotherapies such as adalimumab are considered high-risk groups for whom treatment should be considered [17]. 

**Table 2:** Categories of immunocompromise and risk for disseminated/severe histoplasmosis Taken from the 2025 clinical practice guideline update by the Infectious Diseases Society of America on histoplasmosis: treatment of asymptomatic histoplasma pulmonary nodules (histoplasmomas) in adults, children, and pregnant people [16] AD HIES, autosomal dominant hyperimmunoglobulin E syndrome; NEMO, nuclear factor-kappa B essential modulator; SCID, severe combined immunodeficiency.

High risk	Moderate risk	Low risk
Receiving corticosteroids: ≥2 mg/kg/day of prednisone (or equivalent) for persons <10 kg or ≥20 mg/day of prednisone (or equivalent) for persons >10 kg for at least two weeks	Receiving corticosteroids: 0.5-2 mg/kg/day of prednisone (or equivalent) for persons <10 kg or 5-20 mg/day of prednisone (or equivalent) for persons >10 kg for at least four weeks	Receiving corticosteroids: <0.5 mg/kg/day of prednisone (or equivalent) for persons <10 kg or ≤5 mg/day of prednisone (or equivalent) for persons >10 kg for at least four weeks
Primary cellular immunodeficiency (e.g., SCID, autosomal dominant hyper IgE syndrome (AD HIES), and interferon-gamma receptor/IL-12 pathway defects)	Primary immunodeficiency (e.g., common variable immunodeficiency, nuclear factor kappa B pathway defects (NEMO), chronic mucocutaneous candidiasis, X-linked hyper IgM syndrome, and autosomal recessive HIES)	
Advanced or untreated HIV/AIDS (CD4 <200 cells/mm^3^)	HIV (CD4 200-300 cells/mm^3^)	HIV (CD4 ≥300 cells/mm^3^); viral load undetectable
Hematopoietic stem cell transplant within 100 days or receiving immunosuppressive therapy for graft vs. host disease	Hematopoietic stem cell transplant >100 days prior and no evidence of graft vs. host disease	
Chimeric antigen receptor T-cell therapy within 90 days	Chimeric antigen receptor T-cell therapy >90 days and resolved cytopenias	
Solid organ transplant and treatment of rejection	Solid organ transplant recipient on maintenance immunosuppressive regimen	
Autoimmune and rheumatic diseases requiring treatment with biologic agents, especially those that interfere with T-cell function and granuloma formation		Autoimmune and rheumatic diseases not requiring treatment

The recommended infectious screening tests before initiating a tumor necrosis factor-alpha (TNF-α) inhibitor are for latent tuberculosis and hepatitis B infection. However, studies conducted in North American populations before screening was standard of care determined that histoplasmosis was a more commonly reactivated disease than tuberculosis [18]. Furthermore, recent meta-analyses indicate histoplasmosis may even be more prevalent than once thought [19]. We hence advocate for the future study and consideration of *Histoplasma* screening either before or during treatment. There does not appear to be a correlation with the indication of therapy and hazard of fungal infection, inferring the disease process being treated is unlikely to be a contributing factor [20]. Therefore, it is to be expected that as the breadth of indications increases, so will the frequency of fungal infections. While the current geographic variance of TNF-α inhibitors favors high-income, developed countries, the global usage has universally increased [21]. As availability expands to areas with higher risk for *Histoplasma*, the relative risk ratio associated with TNF-α inhibitors will likely expand as well. The value of extensive pretreatment infectious evaluation and early surveillance will be further elucidated with time.

## Conclusions

This case illustrates the growing prevalence of *Histoplasma capsulatum* infections that strongly correlate with the increase in usage of biologic therapies. It underscores the importance of no longer thinking of *Histoplasma capsulatum* as endemic to only isolated portions of the world. With early identification, clinical remission can be achieved, and essential biologic therapy can be safely resumed. Before initiating a biological agent, the risk of fungal infection must be considered. With regards to current guidelines in asymptomatic histoplasmosis and the high rate of infection with the use of biologic agents, screening for asymptomatic fungal infections and pretreatment before starting these therapies should be further investigated.
